# Portrayal of video-assisted mediastinoscopic lymphadenectomy’s range subsequent to its simultaneous use with uniportal VAT-lobectomy for left-sided NSCLC: a case-based perspective

**DOI:** 10.1186/s13019-023-02277-3

**Published:** 2023-04-17

**Authors:** Marc Hartert, Martin Huertgen

**Affiliations:** Department of Thoracic Surgery, Katholisches Klinikum Koblenz-Montabaur, Rudolf-Virchow-Str. 7-9, 56073 Koblenz, Germany

**Keywords:** Non-small cell lung carcinoma (NSCLC), Mediastinal staging, Mediastinal lymph node dissection, Video-assisted mediastinoscopic lymphadenectomy (VAMLA), Uniportal VAT lobectomy, Pneumothorax

## Abstract

**Background:**

Video-assisted mediastinoscopic lymphadenectomy (VAMLA) is the most precise approach combining staging and therapeutic interventions in non-small cell lung cancer (NSCLC). In the case of left-sided NSCLC, the likelihood of mediastinal lymph node metastases depends on the involvement of the left lung regional lymphatic network. As such, it appears obvious – at least for selected patients with mediastinal staging by either PET-CT or EBUS-TBNA ± EUS-FNA and with cN ≤ 2 – to merge VAMLA and left-sided video-assisted thoracoscopic (VAT) lobectomy for a single-stage therapeutical procedure.

**Case presentation:**

We present the clinical course of an 83-year-old patient following simultaneous VAMLA and VAT-lobectomy for invasive mucinous adenocarcinoma of the left upper lobe with a provisional cT3cN0cM0 stage. The patient developed a clinically relevant postoperative pneumothorax due to a persistent parenchymal air leak. CT scan revealed a substantial pneumomediastinum and showed the capability of VAMLAs range for mediastinal lymph node dissection in a unique way. Following the prompt insertion of a second chest tube, the situation was stabilized with an unremarkable further in-hospital stay. The patient remains free of tumor recurrence or distant metastases at a one-year follow-up.

**Conclusion:**

Presenting this *aperçu*, we encourage reviving the debate on (1) precise mediastinal staging in general and (2) VAMLA’s important role as a diagnostic and therapeutic tool.

## Background

Video-assisted mediastinoscopic lymphadenectomy (VAMLA) is broadly accepted as the most precise combined staging and therapeutic intervention in the context of non-small cell lung cancer (NSCLC) [1–3]. As the regional lymphatic network of the left lung increases the likelihood of mediastinal lymph node metastases, VAMLA is an indispensable tool for left-sided NSCLCs [4–6]. In the case of NSCLCs with an assumed limited expansion (as being staged by either positron emission tomography/computed tomography [PET/CT] or EBUS-TBNA [EndoBronchial UltraSound-guided TransBronchial Needle Aspiration] ± EUS-FNA [Endoesophageal UltraSound Fine Needle Aspiration]) it was obvious to merge VAMLA and left-sided video-assisted thoracoscopic (VAT) lobectomy for a single-stage diagnostic/therapeutic procedure [6]. Unfortunately, the patient described in this case developed a delayed postoperative pneumothorax due to a persistent parenchymal air leak. As it happened, CT scan coincidentally showed a substantial pneumomediastinum, portraying VAMLA‘s outstanding capability of mediastinal lymph node dissection in an impressive and comprehensible way. This case report has been presented in accordance with the Strengthening the Reporting of Observational Studies in Epidemiology (STROBE) guidelines available at www.strobe-statement.org and standard data reporting guidelines [7].

## Case Presentation

**Fig. 1:**
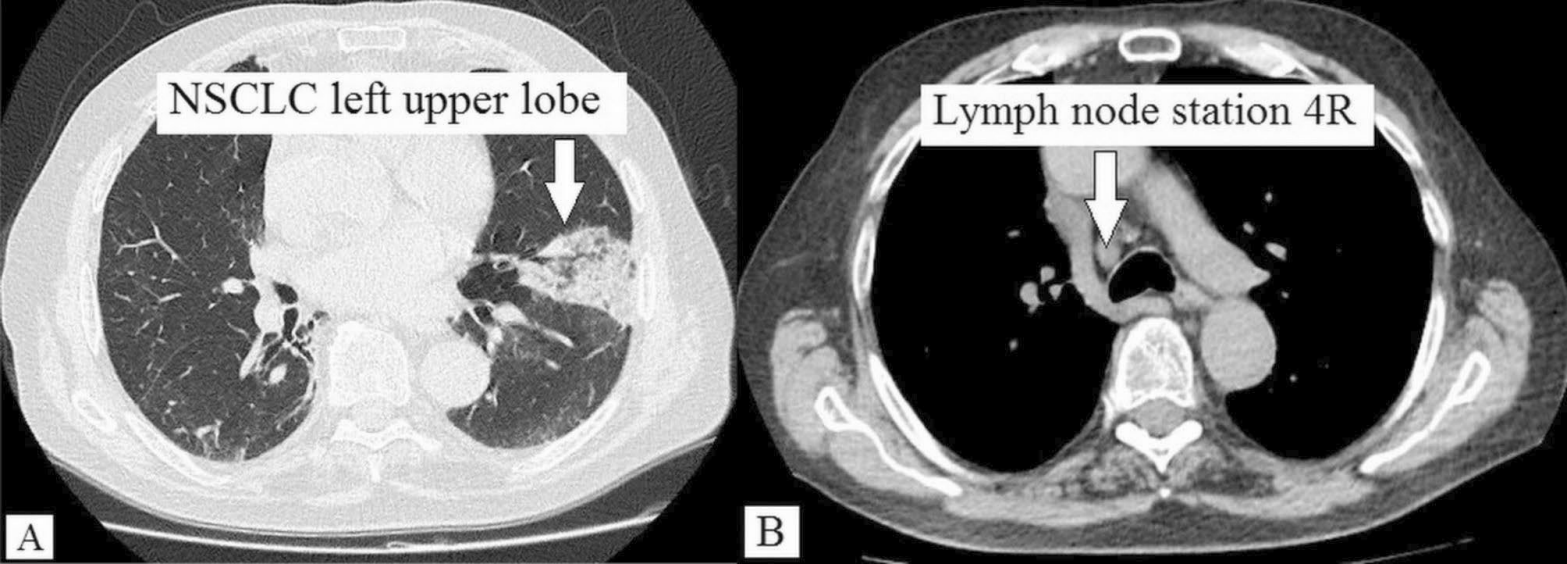
NSCLC of the left upper lobe (A). CT-enlarged mediastinal lymph node station 4R (B)

**Fig. 2:**
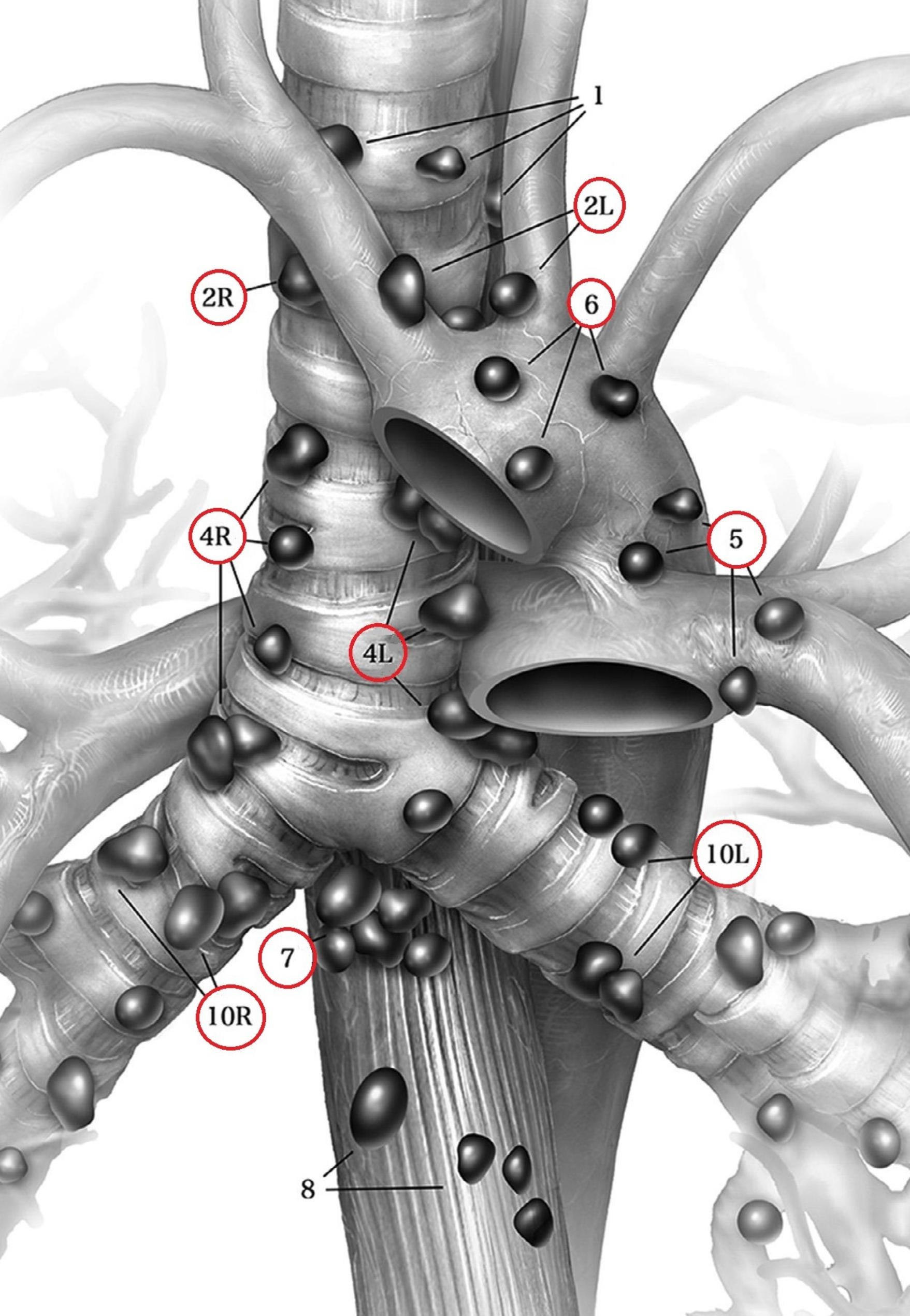
The extent of the resected mediastinal lymph nodes included the following nodal stations: 2L, 2R, 4L, 4R, 5, 6, 7, 10L, and 10R (outlined via red circles) and both 11L and 12L (not part of the illustration; illustration courtesy taken from [3])

**Fig. 3:**
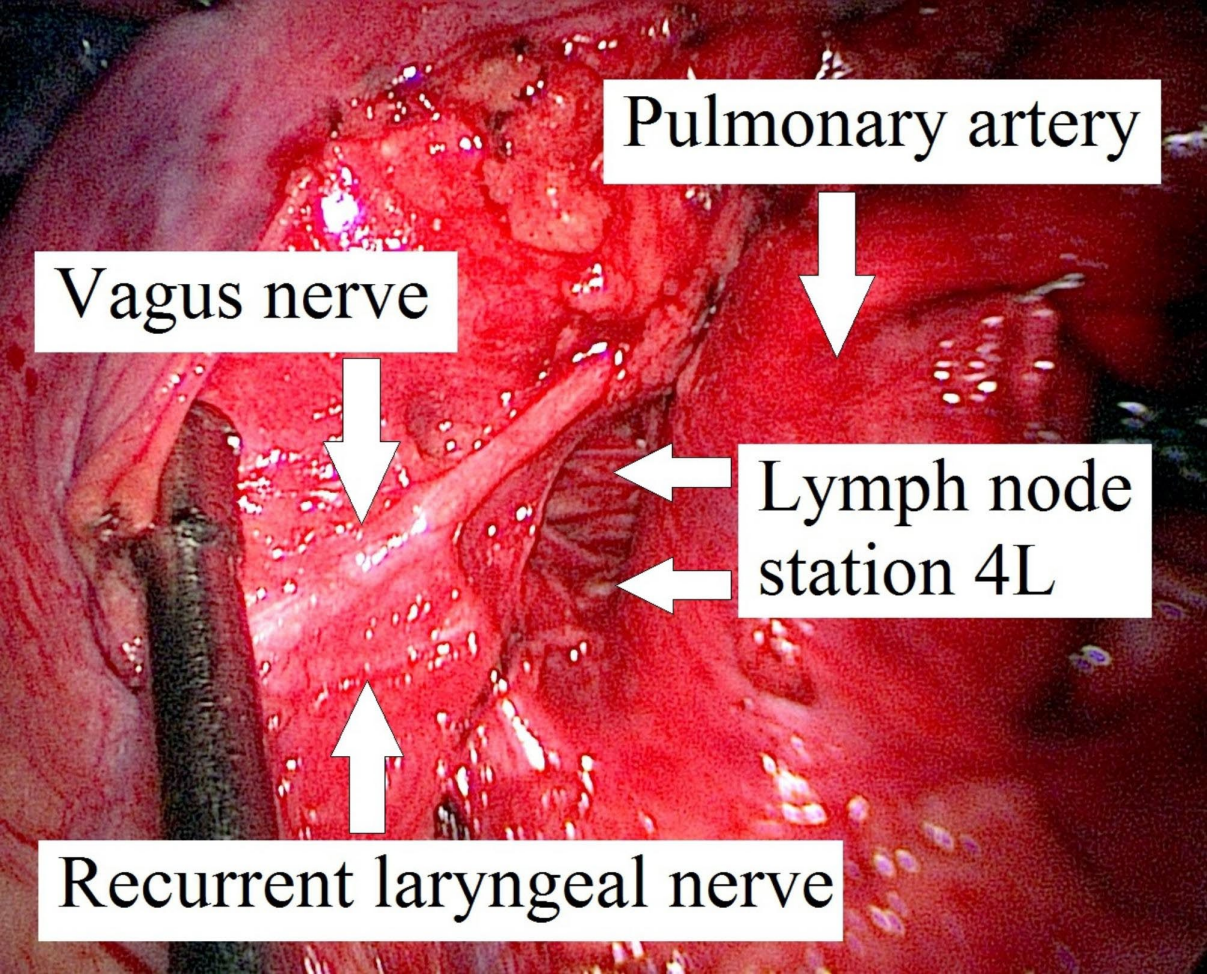
Intraoperative view on the previously VAMLA-dissected lymph node station 4L, taken after having finalized the VAT-resection of the left upper lobe

**Fig. 4:**
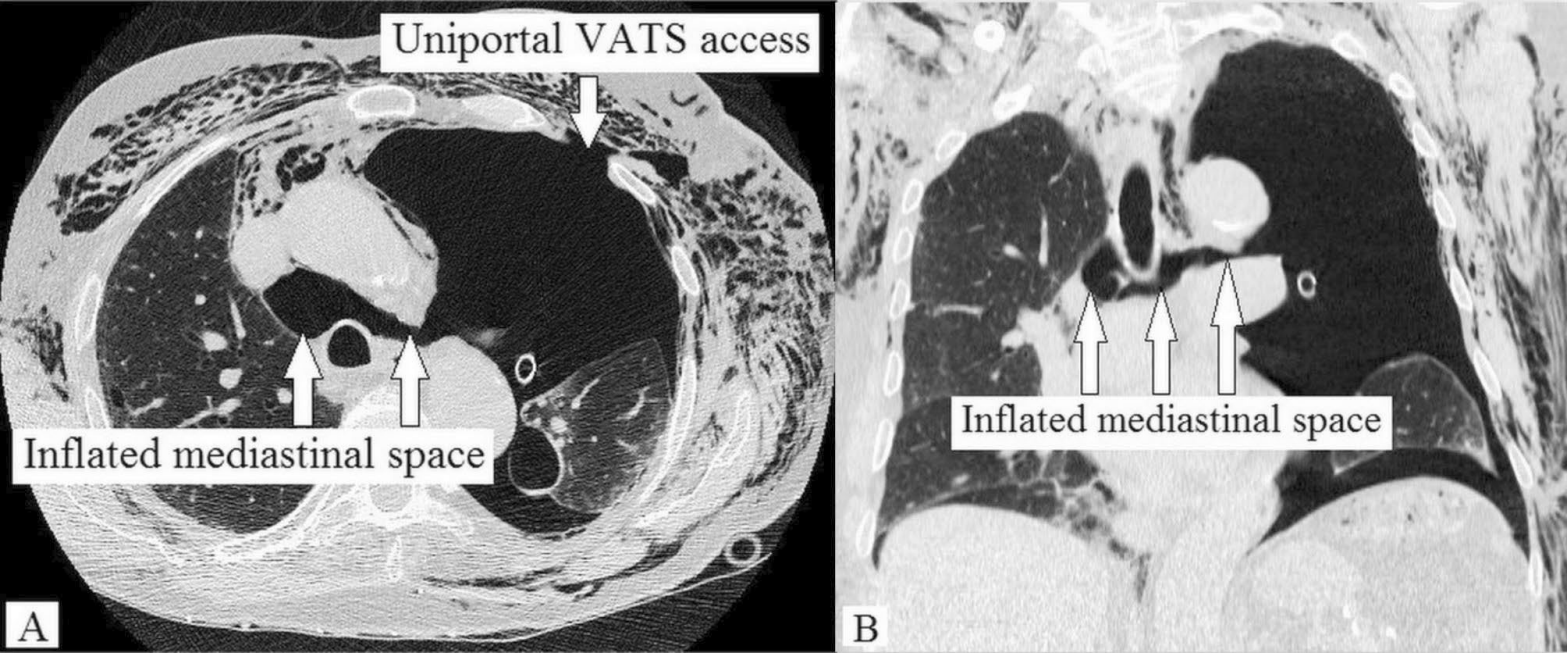
Axial (A) and coronal (B) CT scan showing substantial pneumomediastinum

**Fig. 5:**
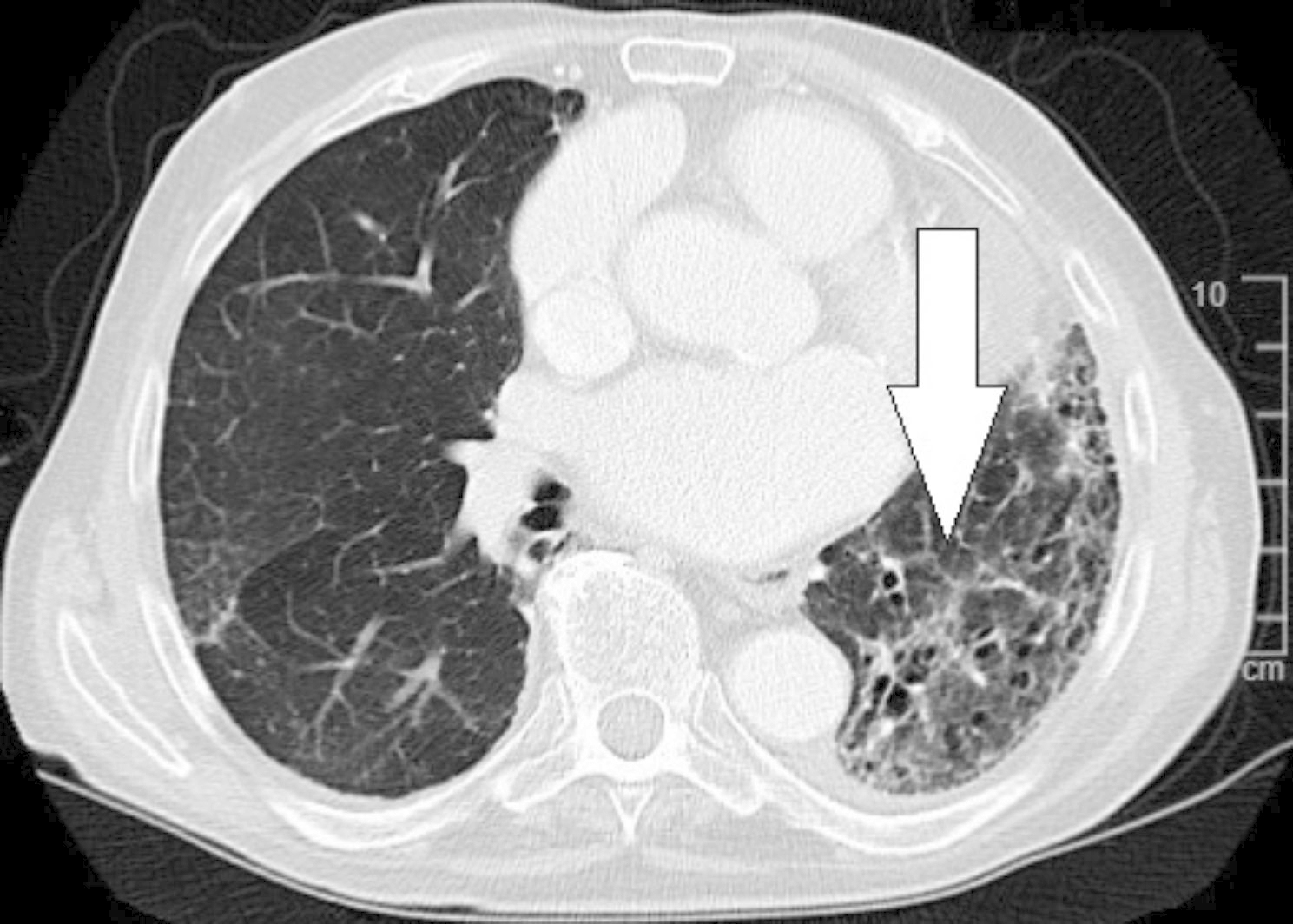
One-year follow-up CT scan excluded short-term tumor recurrence. However, it proved unilaterally pronounced aggravation of fibrotic lung disease in the left lower lobe (arrow)

An 83-year-old non-smoker was diagnosed via transbronchial biopsy with invasive mucinous adenocarcinoma of the left upper lobe (Fig. 1A). Relevant medical history included paroxysmal atrial fibrillation (CHA_2_DS_2_-VASc score 2) and amiodarone-induced fibrotic lung disease (forced vital capacity [FVC]: 77.4%; inspiratory vital capacity [IVC]: 62.7%; forced expiratory volume in 1 s [FEV_1_]: 79.5%). Biomarkers for lung cancer were as follows: cytokeratin 19 fragment antigen 21 − 1 (CYFRA21-1) = 5.06 ng/ml (reference < 3.30 ng/ml), carcinoembryonic antigen (CEA) = 1.5 ng/ml (reference < 5.0 ng/ml), and neuron-specific enolase (NSE) = 14.4 ng/ml (reference < 18.3 ng/ml). 18 F-fluorodeoxyglucose (FDG)-PET/CT excluded mediastinal lymph node and distant metastases. The primary tumor measured 56 × 48 × 39 mm with a maximum standardized uptake value (SUVmax) of 4.5. Provisional TNM staging was cT3cN0cM0. Nevertheless, micro-metastases in the mediastinal lymph nodes could not be excluded with absolute certainty based on (1) the tumor’s biology and (2) CT-enlarged lymph nodes (Fig. 1B). Neither EBUS-TBNA nor EUS-FNA were performed for additional mediastinal staging as the quality of both endoscopic fine-needle aspiration techniques would have been insufficient for systematically mapping the mediastinal lymph node zones. The patient underwent single-stage VAMLA followed by uniportal VAT-lobectomy to achieve an extended mediastinal lymph node dissection. Our surgical VAMLA technique is described in detail elsewhere [8]. In this case, the extent of the resected mediastinal lymph nodes is depicted in Fig. 2. A noteworthy effort of the combined surgical procedure is the repositioning of the patient following VAMLA (carried out in the supine position) for VAT-lobectomy (carried out in the lateral decubitus position). The total operating time (skin-to-skin) for the combined surgical procedure was 215 min (VAMLA: 48 min; repositioning of the patient on the operating table: 33 min; VAT-lobectomy: 134 min). Intraoperative blood loss was negligible (approximately 100-150ml). An intraoperative photograph of the previously VAMLA-dissected lymph node station 4L – taken after having finalized the VAT-resection of the left upper lobe – is shown in Fig. 3. The removed mediastinal lymph nodes did not undergo intraoperative frozen section analysis as (1) a radical lymphadenectomy would have been performed in any case (even with evidence of micro-metastases in N1 lymph nodes), (2) micro-metastases would have been hardly found during the rapid pathological examination and (3) the findings would not have changed the oncologic concept [9]. A few hours postoperatively, the patient developed a delayed pneumothorax due to a persistent parenchymal air leak. The CT scan showed a substantial pneumomediastinum (Fig. 4A, B). The situation was promptly resolved by inserting a second chest tube (Charrière 24) at the 6th intercostal space on the anterior axillary line. The air leak gradually sealed up within eleven days using a digital chest drainage system. Prolonged air leakage is not unusual in patients with fibrotic lung disease. The further in-hospital stay was uneventful. The final histological finding revealed stage IIB invasive mucinous adenocarcinoma (pT3 due to tumor size 52 × 39 × 21 mm, pN0 M0). Considering the patient’s age and N0-status, the tumor board recommended no further therapy as an individual decision. However, close follow-up was mandatory (i.e., CT monitoring in 3-month intervals within the first postoperative year). The patient remains free of tumor recurrence or distant metastases at a one-year follow-up. However, a CT scan confirmed spirometry suspected (FVC: 62.3%; IVC: 49.1%) aggravation of the fibrotic lung disease (Fig. 5).

## Discussion

When we introduced VAMLA over twenty years ago, its continuous value as a mediastinal staging tool was hard to predict [10]. The diagnostic spectrum has substantially broadened as years passed – primarily by different anatomic and metabolic imaging techniques and endoscopies. But the VAMLAs role as the most precise diagnostic mediastinal staging instrument is still unrivaled [11, 12]. For example, endoscopic fine-needle aspiration techniques have their own unique limitations: (1) neither EBUS-TBNA nor EUS-FNA ensures a systematic mapping of the mediastinal lymph node zones as they are limited to individual lymph node samples of interest, and (2) as tumor cells do not consistently affect lymph node tissue, random sampling of lymph nodes may fail to detect cancer cells [13]. For this reason, we did not perform mediastinal staging via one of the endoscopic techniques in this case. Instead, the preoperative staging was carried out via PET-CT, which could exclude mediastinal lymph node metastases.

To quote the famous British surgeon, Sir Berkeley Moynihan (1865–1936): *“the surgery of cancer is not the surgery of organs; it is the surgery of the lymphatic system”*, which remains true to this day [14]. The old quotation emphasizes the importance of modern lymph node dissection via VAMLA. As cancer may spread early via an easy, predictable route within the lymphatic system, precise knowledge of mediastinal lymph node metastasis *and* radical resection of all accessible mediastinal lymph nodes are of particular significance. In principle, the percentage of positive lymph nodes (so called *“node ratio”*) portrays the clinically relevant tumor status more accurately than the strict anatomical N2/3-categorization. This emphasizes the dynamic character of a tumor which is more salient than simply referring to static compartments [15]. Based on this assumption, it is evident that extracting a lymph node via VAMLA or just sampling a lymph node endoscopically makes a considerable therapeutic difference. VAMLA exclusively distinguishes itself due to its additional relevance as a therapeutic intervention and, for this reason, is rightly accepted as a combined staging and therapeutic instrument in the context of lung cancer [8]. As the regional lymphatic pathways of the left lung increase the likelihood of mediastinal lymph node metastases, it is obvious to merge VAMLA and left-sided VAT-lobectomy for a diagnostic and therapeutic one-stage procedure.

The risk-benefit ratio of the most appropriate mediastinal staging instruments has been broadly discussed. Still, the quality and how extensive VAMLA is as a surgical procedure is often underrated. This is not surprising as the extent of mediastinal lymph node dissection can hardly be captured otherwise than by listing dissected lymph node zones and their respective number of resected lymph nodes. In addition, the concept of surgical intervention can be challenging to understand by non-surgeons. The presented CT scans compensate for this deficit by illustrating the radicality of mediastinal lymph node dissection via VAMLA in a unique way – hereby visually highlighting the testimony of Sir Berkeley Moynihan.

To further clarify, notwithstanding these aspects mentioned above, EBUS-TBNA is a highly valuable mediastinal staging tool [16–19]. For example, in one study, the overall diagnostic sensitivity, specificity, accuracy, negative predictive value, and positive predictive value of EBUS-TBNA were 95.7, 100, 97.3, 93.2, and 100%, respectively [16]. Moreover, its staging accuracy was significantly improved when EBUS-TBNA was combined with EUS-FNA [20, 21]. However, neither of the two endoscopic fine-needle aspiration techniques was used in the present case due to PET-CT negative mediastinal lymph nodes. Therefore, the likelihood of relevant mediastinal lymph node metastases was low – if any, identifying skip metastasis would have been a game of chance for EBUS-TBNA. For this reason, we used VAMLA as a therapeutical procedure. VAMLA-extended mediastinal lymph node dissection is a valid tool to detect skip metastasis in contralateral lymph node stations – which is not unusual in the case of left-sided NSCLC [22, 23]. For further consideration: would anyone question the extent of lymph node dissection for sole VAT-lobectomy? Would anyone miss the opportunity to dissect contralateral mediastinal lymph nodes if technically possible? These are essential questions that we avoid directly answering due to the limitations of this case-based perspective. It’s been over twenty years since we introduced VAMLA. We have not yet analyzed our large VAMLA cohort by comparing the long-term survival of the VAMLA cohort to a similar EBUS-TBNA cohort. Proving a presumed longer overall survival of the former would substantially reinforce the key point of this case report.

In summary, the advantages of simultaneous VAMLA and left-sided VAT-lobectomy are: (1) both surgical interventions are indicated; (2) single anesthesia; (3) VAMLA is a *one-surgeon* procedure; (4) VAMLA’s combined diagnostic and therapeutic value; (5) VAT-lobectomy is technically easier in the absence of VAMLA-related mediastinal adhesions; (6) *sloppy lymphadenectomy* following a single VAT-lobectomy is avoided; (7) lymph node dissection is more accurate than a two-stage approach, where the single procedure relies on another and might remain incomplete; (8) equivalent morbidity compared to the two-stage approach; (9) optimized use of OR facilities; (10) quicker completion of the overall oncologic intervention.

## Conclusion

Postoperative diagnostic CT scans portrays the extensive capability of VAMLA when simultaneously used with left-sided VAT-lobectomy in this novel setting to achieve an exceptional approach of extending mediastinal lymph node dissection. Therefore, we encourage reviving the debate on (1) precise mediastinal staging in general and (2) VAMLA’s prominent role – besides its diagnostic impact – as a therapeutic tool in particular.

## Data Availability

Materials of the current study are publicly available from the corresponding author on reasonable request.
